# Workplace-based integrative care for firefighters: real-world evidence from a visiting healthcare program

**DOI:** 10.3389/fpubh.2026.1880013

**Published:** 2026-09-15

**Authors:** Inae Youn, Jisu Ha, Sunjoong Kim, Sohyeon Ryu, Juchul Kim, Ji Hun Wi, Dong Kyun Ko, Sung-Youl Choi, Unho Yang, Jungtae Leem

**Affiliations:** 1 Department of Acupuncture and Moxibustion, National Medical Center, Seoul, Republic of Korea; 2 Department of Clinical Korean Medicine, Graduate School, Kyung Hee University, Seoul, Republic of Korea; 3 Department of Internal Medicine of Korean Medicine, National Medical Center, Seoul, Republic of Korea; 4 Research Institute of Korean Medicine Policy, The Association of Korean Medicine, Seoul, Republic of Korea; 5 Seoul Korean Medicine Association, Seoul, Republic of Korea; 6 Department of Neuropsychiatry, College of Korean Medicine, Gachon University, Seongnam, Republic of Korea; 7 Department of Korean Medicine, Graduate School, Kyung Hee University, Seoul, Republic of Korea; 8 Department of Il-won Integrated Medicine, Wonkwang University Korean Medicine Hospital, Iksan, Republic of Korea; 9 Research Center of Traditional Korean Medicine, College of Korean Medicine, Wonkwang University, Iksan, Republic of Korea

**Keywords:** essential workers, healthcare access, integrative care, musculoskeletal pain, program evaluation, workplace health

## Abstract

**Introduction:**

Firefighters, as essential workers, face substantial barriers to healthcare access due to shift work, unpredictable schedules, and physically demanding duties. Workplace-based healthcare delivery models have emerged as a potential strategy to address these access gaps, yet real-world evidence remains limited.

**Methods:**

This study evaluated a workplace-based visiting healthcare program implemented at fire stations in Seoul, Korea, between September 2023 and February 2024. A retrospective chart review assessed changes in pain intensity among firefighters receiving integrative care, including acupuncture and Chuna manual therapy, while a cross-sectional survey examined user satisfaction, perceived need, and acceptability of the program.

**Results:**

Forty firefighters were included in the clinical evaluation, showing significant reductions in pain intensity (Numeric Pain Rating Scale, median [IQR]: 4.0 [4.0–6.0] to 2.0 [1.0–2.0]; Wilcoxon signed-rank test, *p* < 0.001), with 97.5% achieving the minimal clinically important difference. The intervention was well-tolerated, with only minor and transient adverse events reported. Survey responses from 133 participants indicated high satisfaction (median 4 out of 5 for all items) and strong willingness to recommend the service (88.0%). Most respondents recognized the need for such services (92.0%) and supported their integration into institutional healthcare systems (84.0%).

**Conclusion:**

Workplace-based integrative care may represent a feasible and acceptable model to improve healthcare access among shift-based essential workers. These findings provide real-world evidence supporting workplace-based healthcare delivery models for shift-based essential workers.

## 1 Introduction

Firefighters are continuously exposed to physical and psychological hazards while performing critical duties to protect lives and property, including fire suppression, rescue, and emergency medical services (1). In particular, the prevalence of musculoskeletal disorders has been reported to be markedly higher among firefighters than in the general population owing to heavy equipment use, irregular work schedules, and the challenging environmental conditions at fire scenes (2). These musculoskeletal disorders impair firefighters’ occupational performance and are linked to diminished quality of life and early retirement. Moreover, they have emerged as a major public health issue requiring national-level management (3).

However, healthcare support systems for firefighters remain inadequate. Shift work and on-call duties often limit access to routine healthcare, and the lack of continuity of care after acute-phase treatment frequently results in a chronic course (4). Some firefighters who do not receive adequate pain control may become dependent on self-prescribed analgesics, placing them at higher risk of medication misuse relative to workers in other occupations (5), and a relationship between occupational stress and alcohol use has also been reported (6). Hence, tailored medical support programs that account for firefighters’ unique work environment are needed, including their development and implementation (7).

Korean medicine (KM) has a long history of use for the prevention and treatment of musculoskeletal disorders, and its principal modalities, including acupuncture, pharmacopuncture, and Chuna manual therapy, are widely used for pain relief and functional recovery (8–11). Recent evidence suggests that KM interventions may provide clinically meaningful benefits for various musculoskeletal pain conditions. Meta-analyses of acupuncture trials have reported beneficial effects compared with sham acupuncture across musculoskeletal pain conditions (9), while clinical trials in occupational populations have demonstrated improvements in pain and work-related outcomes among workers with musculoskeletal complaints (12, 13).

Chuna manual therapy has also been evaluated in randomized clinical trials, with studies reporting improvements in pain intensity and disability among patients with chronic neck pain and non-acute low back pain compared with usual care (14, 15). In addition, large-scale retrospective data have suggested that adverse events associated with Chuna manipulation are uncommon and predominantly mild and transient (16). However, evidence for pharmacopuncture remains limited, and the overall evidence base for KM interventions is heterogeneous across modalities and clinical settings (10).

Beyond individual modalities, integrative KM care, including the combination of multiple KM interventions and KM with conventional medical care, has been associated with clinically meaningful improvements in patients with musculoskeletal disorders (17, 18). In Korea, KM treatment is covered by national health insurance (19), and KM practitioners participate in the public health doctor system (20), supporting broad access to KM services. In the occupational health context, workplace-based interventions that minimize time away from duty while providing effective treatment are particularly valuable for essential workers such as firefighters (21). However, evidence regarding the effectiveness and acceptability of workplace-based KM services for firefighters remains limited (7).

Therefore, the Seoul Korean Medicine Association, in collaboration with the Seoul Metropolitan Fire and Disaster Headquarters, implemented a pilot program providing visiting KM services to firefighters from September 2023 through February 2024. Meanwhile, the National Fire Hospital is scheduled to open in June 2026 to provide specialized healthcare services for firefighters (22). The integration of KM within this new healthcare infrastructure could broaden treatment options and enhance access to care for firefighters. However, systematic evaluations of visiting KM services remain scarce, and firefighters’ awareness and specific needs regarding KM services, essential prerequisites for designing firefighter-centered care, have yet to be thoroughly investigated (23).

Therefore, in this study, we aimed to evaluate a workplace-based visiting KM pilot program for firefighters, focusing on its feasibility, acceptability, and potential to improve healthcare access in a shift-based occupational setting.

## 2 Methods

### 2.1 Objectives

The specific objectives of this study were as follows.

(1) To evaluate pain reduction in firefighters with musculoskeletal and other prevalent conditions who participated in the Seoul Metropolitan Visiting KM pilot program.
(2) To assess firefighters’ satisfaction and awareness regarding the above pilot program.
(3) To investigate firefighters’ awareness of and demand for the establishment of KM departments within the National Fire Hospital.
(4) To derive recommendations for the future expansion of KM support for firefighters.

### 2.2 Study design and setting

This multi-method observational study evaluated a visiting KM pilot program funded by the Seoul Metropolitan Council and implemented by the Seoul Korean Medicine Association from 1 September 2023 to 29 February 2024. The program was conducted under a Memorandum of Understanding with the Seoul Metropolitan Fire and Disaster Headquarters and operated across four districts in Seoul. KM practitioners visited individual fire stations once per week to provide clinical services in designated rest areas within the stations. This workplace-based care delivery model was intended to improve healthcare access for firefighters, for whom shift work and standby duties can hinder use of routine healthcare services.

### 2.3 Retrospective chart review for treatment response

Data were collected from a report containing retrospective data for 40 of the 271 firefighters who received treatment through the visiting KM pilot program during a 6-month period (1 September 2023 to 29 February 2024). In the report, cases were excluded from data collection if treatment for musculoskeletal pain was not received or if key information was missing, including the number of visits, chief complaint, treatment details, adverse event status, or pre- and post-treatment evaluations. Because this was an evaluation of a government-funded pilot program, the sample size was determined by program capacity and data completeness rather than by statistical power calculations, which are typically used in pragmatic program evaluation research.

The report included participants’ age, sex, department, first-visit date, chief complaint, treatment records, number of treatment sessions, treatment progress, and baseline pain levels. Chuna, a manual therapy used in East Asian traditional medicine, was primarily administered for each chief complaint with concurrent acupuncture treatment.

### 2.4 Survey for participants

A survey was conducted with the cooperation of the National Fire Agency, targeting 271 firefighters who received treatment through the 6-month visiting KM pilot program. Unlike the retrospective chart review, which applied additional eligibility criteria for evaluation of clinical outcomes, the survey was distributed to all program participants to assess their experiences and perceptions of the service. A survey request was submitted via official notice. The survey was conducted over 4 days, from 26 September to 30 September 2024, and 133 respondents completed it. Based on questionnaires used in previous studies, survey items were developed regarding overall satisfaction with the pilot program and KM treatment progress, necessity, and demand. Specifically, the survey consisted of four sections: (1) general experience and awareness of KM; (2) experience and patterns of use of visiting KM services; (3) satisfaction with and intention to use visiting KM services; and (4) the need to establish a KM department at the National Fire Hospital. After drafting the initial questionnaire items, content and face validity were reviewed by four experts in the fields of clinical practice, health policy, and public medicine, including a university professor specializing in research methodology, a healthcare professional at a public KM institution, a researcher at a KM policy research institute, and an officer of the Korean Medicine Association, to ensure objectivity and enhance the validity of the survey items. The survey comprised items estimated to require approximately 5–10 min. The survey was administered online via Google Forms^1^, and firefighters who participated in the KM pilot program were compensated with a coffee coupon valued at approximately South Korean Won 20,000.

Evaluative items comprised two distinct formats. The six satisfaction items (overall satisfaction and five domain-specific items covering administrative procedures, treatment facilities, treatment effect, staff attitude, and accessibility) were rated on a 5-point Likert-type scale anchored at 1 = “very dissatisfied” and 5 = “very satisfied,” with 3 = “neutral” as an explicit midpoint. The six opinion items—trust in the therapeutic effects of KM, perceived need for KM treatment, agreement that the absence of a KM department limits patients’ treatment choice, expected improvement in patient satisfaction with a KM department, and support for establishing KM departments at the National Fire Hospital and support for establishing KM departments at other public hospitals—were rated on a 4-point forced-choice scale without a neutral midpoint, together with a separate, non-substantive “do not know” option offered as a fifth response category. Because “do not know” does not lie on the underlying ordinal continuum, these responses (ranging from 4.5 to 13.5% across items) were excluded from the ordinal summary and comparative analyses of the corresponding item and are reported separately as a frequency. All evaluative items were treated as ordinal variables in the analysis (see Section 5).

### 2.5 Statistical analysis

Variables were classified into three measurement levels prior to analysis: nominal categorical (e.g., sex, department, chief complaint), ordinal (e.g., age band, visit-count band, NRS scores, and all Likert-type survey items), and continuous (age, in years). NRS scores and Likert-type survey items were treated as ordinal, consistent with their measurement properties (24), and were analyzed using non-parametric methods throughout.

For the clinical evaluation, NRS scores were summarized using medians and interquartile ranges (IQR). Pre- and post-treatment NRS scores were compared using the Wilcoxon signed-rank test, both overall and within subgroups defined by sex, age band, number of visits, and chief complaint; the Hodges–Lehmann estimator was used to estimate the median within-patient change. Differences in baseline NRS between sexes were assessed using the Mann–Whitney U test. Comparisons of NRS change across three or more groups (age band, visit-count band) were assessed using the Kruskal–Wallis test. Responder status (≥2-point or ≥30% reduction in NRS) was compared between sexes using Fisher’s exact test, given the small expected cell counts.

For analyses of NRS change by chief complaint, a composite patient-level NRS value was used as the basis. When multiple chief complaints were reported, the same NRS change value was duplicated and assigned to each corresponding chief complaint category. This approach may have introduced non-independence across symptom categories and should therefore be interpreted with caution.

For the survey evaluation, ordinal survey items (satisfaction and opinion items) were summarized using medians, modes, and ranges rather than means and standard deviations. For the six survey items that included a non-substantive “do not know” response option, “do not know” responses were excluded from the ordinal summary of that item and are reported separately as a frequency. Categorical and binary survey items were summarized as numbers and percentages and compared using the chi-squared test or Fisher’s exact test as appropriate.

All tests were two-sided, and *p*-values ≤0.05 were considered statistically significant. Statistical analyses were performed using R software version 4.3.0 (R Foundation for Statistical Computing, Vienna, Austria).

### 2.6 Standards and methods for evaluating therapeutic response

This was a single-arm before-and-after comparison study without a control group. Pain intensity was assessed using the 11-point Numeric Pain Rating Scale (NRS), on which respondents rated current pain intensity as an integer from 0 to 10, anchored at 0 = “no pain” and 10 = “worst pain imaginable”; pre-treatment ratings were obtained at the first visit and post-treatment ratings at the final visit of the program period. The NRS was used to quantify the treatment effect for each symptom. Because NRS scores are ordinal, improvement was summarized using medians and interquartile ranges, and pre- and post-treatment values were compared using the Wilcoxon signed-rank test, with the Hodges–Lehmann estimator used to summarize the median within-patient change (see Section 5). All tests were two-sided, and *p*-values ≤0.05 were considered statistically significant. To classify responders and non-responders, the MCID (Minimal Clinically Important Difference) was applied. The MCID represents the smallest change in an outcome score that patients perceive as beneficial (25). Patients with a reduction of 2 or more points or 30% or more in the NRS score were classified as responders, reflecting a clinically meaningful improvement in pain intensity (26). Responder status was compared between sexes using Fisher’s exact test.

### 2.7 Safety evaluation standards and methods

Safety evaluations were performed for all adverse events documented in the charts during the treatment period. Incidence rates were reported for adverse events overall, adverse events leading to withdrawal, and serious adverse events. Adverse events were captured at each visit from patient-reported symptoms and investigator observations.

### 2.8 Analysis of survey results

After survey completion, ordinal survey items were analyzed in R as described in Section 5. For each item, the proportion (%) of each response, together with the median, mode, and range, were reported; for the six opinion items with a “do not know” option, the frequency of “do not know” responses are reported separately. Subgroup analyses were performed by job type, age, and sex.

## 3 Results

### 3.1 Patient and treatment characteristics

The retrospective chart review included 40 participants (55.0% men). The mean participant age was 48.6 ± 10.5 years, and the mean number of visits was 6.9. The most common chief complaint was lower back pain (75.0%), followed by shoulder (62.5%), pelvic (60.0%), and neck pain (57.5%). Median baseline NRS was 4.0 (IQR 4.0–6.0) overall; baseline NRS was significantly higher among men (median 5.0, IQR 4.0–6.0) than women (median 4.0, IQR 4.0–4.0) (Mann–Whitney *U* test, *U* = 269.5, *p* = 0.043). Detailed participant characteristics are presented in Table 1.

**Table 1:** General characteristics of participants (*n* = 40).

Characteristics	Male	Female	Total
Number, *n* (%)	22 (55.0)	18 (45.0)	40
Age, mean ± SD	49.2 ± 9.0	47.2 ± 11.9	48.6 ± 10.5
Number of visits, mean ± SD (median)	7.9 ± 3.50 (7.5)	5.6 ± 3.78 (5.0)	6.9 ± 3.76 (6.0)
Department, *n* (%)			
Fire administration division	5 (22.7)	14 (77.7)	19 (47.5)
Field response unit	7 (31.8)	3 (16.7)	10 (25.0)
Prevention division	5 (22.7)	1 (5.6)	6 (15.0)
Disaster management division	4 (18.2)	0 (0.0)	4 (10.0)
119 safety center	1 (4.6)	0 (0.0)	1 (2.5)
Chief complaints, *n* (%)			
Low back pain	16 (72.7)	14 (77.8)	30 (75.0)
Pelvic pain	13 (59.1)	11 (61.1)	24 (60.0)
Shoulder pain	12 (54.5)	13 (72.2)	25 (62.5)
Neck pain	14 (63.6)	9 (50.0)	23 (57.5)
Knee pain	1 (4.6)	3 (16.7)	4 (10.0)
Upper limb numbness	3 (13.6)	1 (5.6)	4 (10.0)
Finger/wrist pain	0 (0.0)	1 (5.6)	1 (2.5)
Plantar pain	1 (4.6)	0 (0.0)	1 (2.5)
Other baseline information, *n* (%)			
Lumbar disc/lumbar stenosis	7 (31.8)	9 (50.0)	16 (40.0)
Pelvic disorder	1 (4.6)	0 (0.0)	1 (2.5)
Shoulder disorder	1 (4.6)	2 (11.1)	3 (7.5)
Cervical disc	6 (27.3)	3 (16.7)	9 (22.5)
Knee disorder	1 (4.6)	3 (16.7)	4 (10.0)
Scoliosis	1 (4.6)	0 (0.0)	1 (2.5)
Plantar fasciitis	1 (4.6)	0 (0.0)	1 (2.5)
No information	5 (22.7)	4 (22.2)	9 (22.5)
Initial NRS (pre-treatment NRS), median (IQR)			
NRS	5.0 (4.0–6.0)	4.0 (4.0–4.0)	4.0 (4.0–6.0)

KM, Korean medicine; NRS, Numeric Pain Rating Scale; SD, standard deviation; IQR, interquartile range.

### 3.2 Treatment effect

Median NRS scores decreased significantly from 4.0 (IQR 4.0–6.0) pre-treatment to 2.0 (IQR 1.0–2.0) post-treatment (Wilcoxon signed-rank test, *p* < 0.001). The median within-patient change was 3.0 (IQR 2.0–4.0; Hodges–Lehmann estimate 3.0, 95% CI 2.5–3.0), with post-treatment scores falling to two points or below for most patients (Table 2).

**Table 2:** Comparison of NRS before and after treatment using the Wilcoxon signed-rank test (*n* = 40).

Group	*n* (%)	Pre-treatment NRS median (IQR)	Post-treatment NRS median (IQR)	Change NRS median (IQR)	Wilcoxon W	*p*-value
Total	40 (100.0%)	4.0 (4.0–6.0)	2.0 (1.0–2.0)	3.0 (2.0–4.0)	0.0	<0.0001
Sex						
Men	22 (55.0%)	5.0 (4.0–6.0)	2.0 (1.0–2.0)	3.0 (2.0–4.0)	0.0	<0.0001
Women	18 (45.0%)	4.0 (4.0–4.0)	2.0 (1.0–2.0)	2.0 (2.0–3.0)	0.0	0.0001
Age group						
20–39 years	9 (22.5%)	4.0 (3.0–6.0)	1.0 (1.0–2.0)	3.0 (2.0–4.0)	0.0	0.0039
40–59 years	25 (62.5%)	4.0 (4.0–6.0)	2.0 (1.0–2.0)	3.0 (2.0–4.0)	0.0	<0.0001
≥60 years	6 (15.0%)	4.0 (4.0–5.5)	2.0 (1.2–2.0)	2.5 (2.0–3.0)	0.0	0.0312
Number of visits						
<5 visits	12 (30.0%)	4.0 (3.8–5.2)	1.5 (1.0–2.0)	2.5 (2.0–3.2)	0.0	0.0005
5–10 visits	20 (50.0%)	4.0 (4.0–5.2)	2.0 (1.0–2.0)	2.5 (2.0–3.2)	0.0	<0.0001
>10 visits	8 (20.0%)	5.5 (4.0–6.2)	2.0 (1.8–2.2)	3.0 (2.8–4.0)	0.0	0.0078
Chief complaint						
Lower back pain	30 (75.0%)	4.0 (4.0–5.8)	2.0 (1.0–2.0)	3.0 (2.0–4.0)	0.0	<0.0001
Pelvic pain	24 (60.0%)	4.0 (4.0–6.0)	2.0 (1.0–2.0)	3.0 (2.0–4.0)	0.0	<0.0001
Shoulder pain	25 (62.5%)	4.0 (4.0–6.0)	2.0 (1.0–2.0)	2.0 (2.0–3.0)	0.0	<0.0001
Neck pain	23 (57.5%)	4.0 (4.0–5.5)	2.0 (1.0–2.0)	3.0 (2.0–4.0)	0.0	<0.0001
Knee pain	4 (10.0%)	5.0 (4.0–6.0)	2.0 (2.0–2.2)	2.5 (2.0–3.2)	0.0	0.1250
Upper limb numbness	4 (10.0%)	5.5 (4.8–6.2)	2.5 (2.0–3.0)	3.0 (2.8–3.2)	0.0	0.1250
Wrist/finger pain	1 (2.5%)	6.0	1.0	5.0	—	—
Plantar pain	1 (2.5%)	4.0	0.0	4.0	—	—

NRS, Numeric Pain Rating Scale; IQR, interquartile range.

Statistically significant pain reductions (Wilcoxon signed-rank test, *p* < 0.05) were observed across all subgroups defined by sex, age band, and number of visits, and across chief-complaint subgroups with *n* ≥ 13. For the two smallest chief-complaint subgroups (knee pain and upper limb numbness, *n* = 4 each), the median improvement was comparable in magnitude to the larger subgroups, but the exact Wilcoxon test could not reach statistical significance at this sample size (*p* = 0.125 for each), reflecting limited statistical power rather than an absence of clinical effect.

Across chief-complaint subgroups with adequate sample size (*n* ≥ 13: low back, neck, pelvic, and shoulder pain), NRS scores decreased significantly from pre- to post-treatment (Wilcoxon signed-rank test, *p* < 0.001 for each), with a similar median improvement of 3.0 points for low back, neck, and pelvic pain, and a median improvement of 2.0 points for shoulder pain. As described in the Methods section, in patients reporting multiple chief complaints, the same NRS value was duplicated and entered under each relevant symptom category.

Overall, 97.5% of patients were classified as responders, with response rates of 95.5% in men and 100% in women.

Mild adverse events were reported in three patients (7.5%): temporary soreness at the acupuncture sites (*n* = 2) and transient fatigue (*n* = 1). All events resolved spontaneously on the same day without intervention.

### 3.3 Participants’ satisfaction

A total of 133 firefighters participated in the survey, of whom 78.9% were men and 21.1% were women. In the Korean fire service system, firefighters comprise not only frontline firefighting personnel but also emergency medical, rescue, administrative, and other support staff working within fire departments. Firefighting personnel accounted for the largest proportion of respondents (75.2%; Table 3).

**Table 3:** General characteristics of the survey respondents (*n* = 133).

Category	Item	Frequency (*n*)	Percentage (%)
Sex	Men	105	78.9
	Women	28	21.1
Age, years	20s	7	5.3
	30s	38	28.6
	40s	42	31.6
	50s	44	33.1
	60s or older	2	1.5
Work experience	<10 years	41	30.8
	10–20 years	44	33.1
	20–30 years	29	21.8
	≥30 years	19	14.3
Job type	Firefighting	100	75.2
	Rescue	7	5.3
	Emergency medical	11	8.3
	Driving	10	7.5
	IT	1	0.8
	Communications	2	1.5
	Other (fire investigation)	2	1.5
Rank (ascending rank)	Firefighter	10	7.5
	Senior firefighter	18	13.5
	Fire sergeant	37	27.8
	Fire lieutenant	44	33.1
	Fire captain	16	12.0
	Fire major	8	6.0
Number of visits (pilot program)	1 time	53	39.8
	2 times	29	21.8
	3 or more times	51	38.3

IT, information technology.

#### 3.3.1 Results of KM awareness and experience items

Among respondents, 70.7% reported visiting KM institutions within the past year, and 82.0% stated that they trusted the therapeutic effects of KM. Regarding the preferred healthcare institution, the most common response was “depends on the disease” (54.1%; Table 4).

**Table 4:** Survey results on KM awareness and experience among firefighters (*n* = 133).

Survey question	Response options	Number of respondents	Response rate (%)
1. Experience using KM institutions in the past year	Yes	94	70.68
	No	39	29.32
2. Usually preferred medical institution	KM (clinic, KM hospital)	13	9.77
	WM (clinic, hospital)	39	29.32
	Depends on the disease	72	54.14
	No particular preference	8	6.02
	Do not know	1	0.75
3. Trust in the therapeutic effects of KM	Do not know	6	4.51
	Do not trust at all	0	0.00
	Do not trust much	18	13.53
	Trust somewhat	82	61.65
	Trust very much	27	20.30

KM, Korean medicine; WM, western medicine.

For the ordinal item “Trust in the therapeutic effects of KM” (excluding “do not know” responses, *n* = 127, 4.5% excluded), the median rating was 3 (“trust somewhat”), the modal rating was 3, and the range was 2–4.

#### 3.3.2 Results of items related to the visiting KM service for firefighters

Among participants, 49.6% received treatment for lower back pain, followed by shoulder (33.1%) and neck pain (30.1%). Regarding treatment modality, Chuna manual therapy alone was the most common (45.1%), followed by Chuna manual therapy combined with acupuncture (33.1%) and acupuncture alone (21.8%).

Overall, 88% of respondents reported a willingness to recommend the service to others, with a significant difference by sex (Fisher’s exact test, *p* = 0.023). Willingness to recommend was 100.0% among women and 84.8% among men. The most frequently cited reasons for recommending the service were the convenience of the visiting-care format (65.8%) and treatment effectiveness (55.6%).

For all six satisfaction items, the median rating was 4 on the 5-point scale (range 1–5); the modal rating was 5 for healthcare provider’s attitude and accessibility/convenience, and 4 for the remaining items. Adverse events were reported by two participants (1.5%) (Table 5). No serious adverse events were reported.

**Table 5:** Survey results on visiting KM service for firefighters (*n* = 133).

Survey question	Response options	Response/score
Treatment methods received (*n* = 133)	Chuna only	60 (45.1%)
	Acupuncture only	29 (21.8%)
	Both acupuncture and Chuna	44 (33.1%)
Symptoms treated (*n* = 133, multiple responses were allowed)	Lower back pain	66 (49.6%)
	Shoulder pain	44 (33.1%)
	Neck pain	40 (30.1%)
	Other symptoms	27 (20.3%)
Service recommendation (*n* = 133)	Willingness to recommend the service (yes)	117 (88%)
	Willingness to recommend the service (no)	16 (12%)
Reasons for recommendation (*n* = 117, multiple responses were allowed)	Convenient visiting format	77 (65.8%)
	Good treatment effectiveness	65 (55.6%)
	Detailed explanations provided	26 (22.2%)
	Few side effects	9 (7.7%)
	Other reasons	1 (0.9%)
Adverse events (*n* = 133)	Experience of adverse events: yes	2 (1.50%)
	Experience of adverse events: no	131 (98.5%)
Satisfaction scores (5-point scale)	Healthcare provider’s attitude	4 (mode 5; range 1–5)
	Accessibility/convenience	4 (mode 5; range 1–5)
	Overall satisfaction	4 (mode 4; range 1–5)
	Administrative procedures	4 (mode 4; range 1–5)
	Treatment effectiveness	4 (mode 4; range 1–5)
	Treatment process	4 (mode 4; range 1–5)

KM, Korean medicine; NRS, Numeric Pain Rating Scale; Chuna, Chuna manual therapy.

#### 3.3.3 Results of items related to the establishment of a KM department in the national fire hospital

Although 67.7% of respondents were aware of the establishment of the National Fire Hospital, only 8.3% were aware that the KM department was not included. Overall, 92.0% agreed on the necessity of KM treatment for firefighters, and 86.0% expressed the view that patient satisfaction would improve with the establishment of a KM department within the National Fire Hospital. Support for establishing a KM department in the National Fire Hospital was expressed by 84.0% of respondents, and 84.0% also supported establishing KM departments in other public medical institutions, such as the National Police Hospital and veterans’ hospitals. Regarding expectations from the National Fire Hospital, high-level rehabilitation treatment (73%) ranked highest, followed by medical expense support (67%) and a wide range of medical departments (52%) (Table 6).

**Table 6:** Survey results on the national fire hospital and KM department (*n* = 133).

Category	Item	Response (%)
Awareness of the national fire hospital establishment	Yes	90 (67.7%)
	No	43 (32.3%)
Awareness of the KM department exclusion	Yes	11 (8.3%)
	No	122 (91.7%)
Opinions on KM (4-point scale, “do not know” excluded)	KM treatment is necessary for firefighters (Somewhat + Very much agree)	122 (92%) agree; median 4, mode 4, range 2–4 (*n* = 125; 6.0% “do not know” excluded)
	Excluding the KM department restricts patient choice (Somewhat + Very much agree)	97 (73%) agree; median 3, mode 3, range 1–4 (*n* = 117; 12.0% “do not know” excluded)
	Establishing a KM department improves patient satisfaction (Somewhat + Very much agree)	115 (86%) agree; median 3, mode 3, range 1–4 (*n* = 123; 7.5% “do not know” excluded)
	Support the establishment of the KM department in the National Fire Hospital (Somewhat + Very much agree)	111 (84%) agree; median 3, mode 3, range 1–4 (*n* = 118; 11.3% “do not know” excluded)
	Support the establishment of the KM department in police/veterans hospitals (Somewhat + Very much agree)	112 (84%) agree; median 3, mode 3, range 2–4 (*n* = 115; 13.5% “do not know” excluded)
Expectations for national fire hospital (multiple responses were allowed)	High-level rehabilitation treatment	97 (73%)
	Medical expense support	89 (67%)
	Diverse medical departments	69 (52%)
	Burn and special treatment	52 (39%)
	Post-operative care	48 (36%)
	Other	5 (4%)

KM, Korean medicine.

## 4 Discussion

### 4.1 Summary of study findings

This study provides real-world evidence that workplace-based integrative care can serve as a feasible and acceptable approach to improving healthcare access among firefighters, a population facing substantial structural barriers due to shift-based work. While clinically meaningful reductions in pain were observed, the primary contribution of this study lies in demonstrating the real-world feasibility and practicality of delivering healthcare services directly within occupational settings. This model may help overcome access limitations associated with time constraints, irregular schedules, and delayed care-seeking behaviors commonly observed in essential workers.

Clinically meaningful improvements in pain intensity were observed, as reflected by reductions in NRS scores (median [IQR]: 4.0 [4.0–6.0] to 2.0 [1.0–2.0], Wilcoxon signed-rank test, *p* < 0.001), a median change of 3.0 points (IQR 2.0–4.0), and a high MCID achievement rate (97.5%), suggesting potential clinical benefit. Adverse events were mild. Survey results indicated that 88.0% were willing to recommend the service to others, and the median rating was 4 out of 5 across all satisfaction items. Furthermore, 92.0% recognized the need for KM treatment for firefighters, and 84.0% supported the broader integration of such services into occupational healthcare systems.

### 4.2 Comparison with prior studies and implications for workplace-based care

According to international studies, firefighters have a markedly higher prevalence of musculoskeletal disorders than the general population (27, 28), and domestic studies have similarly reported that firefighters are exposed to a range of musculoskeletal disorders because of the nature of their occupation (29). In this study, the prevalence rates of lower back pain, shoulder pain, and neck pain were 75.0, 62.5, and 57.5%, respectively, which is consistent with findings from previous studies (30).

Whereas previous studies have largely focused on estimating prevalence and conducting epidemiological analyses, this study differed by applying KM interventions to assess actual treatment effects and by proposing practical solutions via an on-site, visit-based care model. This approach can be considered a practical implementation of recommendations from prior studies calling for a health-support system that reflects firefighters’ working environment (21).

As summarized in the Introduction, the effectiveness of acupuncture and Chuna manual therapy for neck and low back pain has been demonstrated in randomized controlled trials (12–15), and the safety of Chuna has been documented in large-scale retrospective data (16). Our findings extend this literature in two respects. First, the observed magnitude of pain reduction and the low rate of mild, self-limiting adverse events are consistent with those trials, but were obtained in a real-world occupational setting rather than a clinical research environment. Second, and in contrast to those trials, the present study evaluated whether such care can be delivered at the workplace to a shift-based essential workforce, and whether the recipients regard that delivery model as acceptable.

The observed median NRS reduction of 3.0 points (IQR 2.0–4.0), MCID achievement rate of 97.5%, and low rate of mild adverse events are consistent with prior evidence, supporting the feasibility and potential effectiveness of this workplace-based care model in a specialized occupational setting. This study is particularly meaningful because treatment effects were evaluated using real-world clinical care data from firefighters, supplementing the limited clinical evidence previously reported in special-category public officials. Additionally, by analyzing both clinical outcomes and satisfaction with, and demand for, visiting care services following the policy pilot program, this study provides empirical evidence applicable to the practical design and institutionalization of healthcare policies for firefighters. Therefore, this work may help bridge the gap between clinical evidence and real-world healthcare delivery in occupational setting.

### 4.3 Implications for occupational health practice and policy

Firefighters often have limited access to healthcare facilities because of work-related factors, including shift work and standby duties (31). These constraints can pose substantial barriers to timely care and may contribute to musculoskeletal condition chronicity (32). Traditional healthcare delivery typically requires workers to leave the workplace and arrange appointments during off-duty hours, further restricting access for shift workers (33). Against this backdrop, the visiting KM care model used in this study provided an alternative delivery approach intended to improve access to care.

By delivering services directly to fire stations, this model allows treatment during working hours without compromising operational readiness, enabling earlier intervention before conditions become chronic. Satisfaction with accessibility and convenience was high (median 4, mode 5, range 1–5), and willingness to recommend the service was strong (88.0%); “the convenience of the visiting care format” was the most frequently cited reason for recommendation (65.8%), supporting the feasibility and acceptability of this delivery model.

Additionally, the clinically meaningful pain reduction observed in this study (97.5% MCID achievement) suggests that workplace-integrated care may have potential value for supporting occupational health. Given the emphasis on preventive approaches and early intervention in prior reviews of firefighter injury profiles (34), an on-site visiting care system may be an effective strategy for reducing long-term disability risk among firefighters.

Finally, previous studies have emphasized the need for institutional support for occupational diseases among special-category public officials, including police officers (35) and firefighters (36). In line with this, our findings suggest that workplace-based integrative care may be a valuable component of broader healthcare support systems for essential workers.

### 4.4 Strengths and limitations of the study

To the best of our knowledge, this study provides the first systematic evaluation of firefighters’ occupational health needs addressed through workplace-based integrative care, using a pragmatic design that yields evidence directly applicable to policy decisions. Strengths include the multi-method observational approach, the use of MCID to support clinical meaningfulness, and real-world implementation, which ensures high ecological validity. Furthermore, this study directly addresses a critical healthcare gap for essential shift workers, providing highly pragmatic evidence that can inform occupational health and safety protocols.

This study has several limitations. First, the absence of a control group limits causal inference regarding treatment effectiveness. Second, the relatively small sample size and single-region setting may affect generalizability. Third, the retrospective design of the clinical evaluation may introduce selection bias. Fourth, regarding the survey outcomes, there was a temporal gap of approximately 7 months between the conclusion of the pilot program and the administration of the survey. This delay may have introduced recall bias, potentially affecting the accuracy of the participants’ self-reported satisfaction and subjective experiences. Finally, although the survey appropriately targeted users of the program to assess direct service satisfaction, their perceptions regarding broader institutional integration (e.g., support for KM departments within the National Fire Hospital) may have been positively overestimated, as the cohort already had favorable prior experiences with the intervention. Therefore, the findings should be interpreted as exploratory and hypothesis-generating, warranting further investigation through larger, controlled studies.

Future research should use randomized controlled trials with longer follow-up periods (3, 6, and 12 months) and larger samples stratified by occupational subgroup, with individual symptom tracking, economic evaluations, and occupational health metrics, to strengthen the evidence base for policy decisions.

## 5 Conclusion

This study demonstrates that workplace-based integrative care is a feasible and acceptable approach for improving healthcare access among firefighters. The significant improvement in NRS scores together with the high MCID achievement rate indicates clinically meaningful benefits. High patient satisfaction and willingness to recommend the service further support its acceptability.

The primary significance of this study lies in identifying the unmet medical needs of a specialized group of essential workers and proposing practical solutions, including improved healthcare access through on-site service delivery and integrated care approaches. The visiting KM care model represents a service delivery approach that may improve access to care for essential workers facing barriers to healthcare access. This study suggests that a workplace-based KM program may provide clinically meaningful benefits and could inform the integration of complementary medicine into occupational health services.

## Data Availability

The data that support the findings of this study are available from the corresponding author upon reasonable request.

## Footnotes

1
https://forms.gle/c4auC2aq49g9jVnw6
